# The effect of canine disimpaction performed with temporary anchorage devices (TADs) before comprehensive orthodontic treatment to avoid root resorption of adjacent teeth

**DOI:** 10.1590/2177-6709.21.2.065-072.oar

**Published:** 2016

**Authors:** Farzin Heravi, Hooman Shafaee, Ali Forouzanfar, Seyed Hossein Hoseini Zarch, Mohsen Merati

**Affiliations:** 1 Professor, Mashhad University of Medical Sciences, Dental Research Center, Department of Orthodontics, School of Dentistry, Mashhad, Iran.; 2 Assistant professor, Mashhad University of Medical Sciences, Oral and Maxillofacial Diseases Research Center, Department of Orthodontics, School of Dentistry, Mashhad, Iran.; 3 Assistant professor, Mashhad University of Medical Sciences, Dental Research Center, Department of Periodontics, School of Dentistry, Mashhad, Iran.; 4 Associate professor, Mashhad University of Medical Sciences, Dental Materials Research Center, Department of Oral and Maxillofacial Radiology, School of Dentistry, Mashhad, Iran.; 5 Assistant professor, Shahed University of Medical Sciences, Tehran, Iran.

**Keywords:** TADs, Impacted canine, CBCT.

## Abstract

**Objective:**

The aim of this study was to evaluate the movement of impacted canines away from the roots of neighboring teeth before full-mouth bracket placement, performed by means of TADs to decrease undesired side effects on adjacent teeth.

**Methods:**

The study sample consisted of 34 palatally impacted canines, being 19 in the experimental group and 15 in the control group. In the experimental group, before placement of brackets, the impacted canine was erupted by means of miniscrews. In the control group, after initiation of comprehensive orthodontics, canine disimpaction was performed by means of a cantilever spring soldered to a palatal bar. At the end of treatment, volume of lateral incisors and canine root resorption were measured and compared by means of a CBCT-derived tridimensional model. Visual Analogue Scale (VAS) score, bleeding on probing (BOP) and gingival index (GI) were recorded. Clinical success rate was also calculated.

**Results:**

The volume of root resorption of lateral teeth in the control group was significantly greater than in the experimental group (*p* < 0.001). At the end of treatment, VAS score, GI and BOP were not significantly different between the two groups.

**Conclusion:**

Based on our results, it seems that disimpaction of canines and moving them to the arch can be done successfully carried out with minimal side effects by means of skeletal anchorage.

## RESUMO

**Objetivo:**

o objetivo do presente estudo foi avaliar o uso de dispositivos de ancoragem temporária (DATs) para a movimentação de caninos impactados, afastando-os das raízes dos dentes vizinhos, antes da colagem dos braquetes em todos os dentes, com o objetivo de minimizar os efeitos colaterais indesejáveis nesses dentes adjacentes.

**Métodos:**

a amostra consistiu de 34 caninos impactados por palatino, sendo 19 no grupo experimental e 15 no grupo controle. No grupo experimental, antes da colagem dos braquetes, os caninos impactados foram tracionados utilizando-se mini-implantes. No grupo controle, após o início do tratamento ortodôntico, a desimpacção dos caninos foi realizada com uma mola em cantiléver soldada a uma barra transpalatina. Ao fim do tratamento, os valores referentes à reabsorção radicular nos incisivos laterais e caninos foram medidos e comparados por meio de modelos tridimensionais reconstruídos a partir de TCFCs. Foram também registrados os escores relativos à dor sentida pelos pacientes, usando uma escala visual analógica (VAS); além do Índice de Sangramento à Sondagem (ISS) e do Índice Gengival (IG). O índice de sucesso clínico também foi calculado.

**Resultados:**

o volume de reabsorção radicular nos incisivos laterais no grupo controle foi significativamente maior do que no grupo experimental (*p* < 0,001). Ao fim do tratamento, não houve diferença significativa entre os dois grupos quanto aos escores relativos à VAS, ao IG e ISS.

**Conclusão:**

esses resultados sugerem que a desimpacção de caninos e a movimentação deles para a arcada dentária podem ser realizadas, com sucesso e com mínimos efeitos colaterais, por meio da ancoragem esquelética.

## INTRODUCTION

Impaction of maxillary permanent canines is a common clinical problem in the dental office.^1^ Ericson and Kurol^2^ reported that the incidence of maxillary canine impaction is 1.7%.^2^ Moreover, it is estimated that the incidence of palatal impaction is two or three times greater than labial impaction.^3^ Maxillary canines play an important aesthetic and functional role; therefore, in this regard, treatment is essential; however, impacted canines are more difficult and time-consuming to treat. Furthermore, impacted canines have a variable axial inclination and location and can lead to resorption of neighboring teeth, especially lateral incisors.^4^

Diagnosis of canine impaction is based on clinical and radiographic evaluations.^5^ Assessing the condition of the lateral incisor root is crucial, since in 80% of cases the roots resorbed by impacted canines were those of lateral incisors.^4^

Two-dimensional imaging modalities can obscure the presence of resorption, and its severity may also be underestimated because of structural superimposition.^6^ However, 3D techniques are more sensitive when compared to 2D methods. The proximity of impacted canines to neighboring lateral incisors can be easily evaluated, both quantitatively and qualitatively by means of 3D imaging, such as cone-beam computed tomography (CBCT).^7^ The use of CBCT improves diagnostic capabilities as well as the chances of success in more difficult cases.^8^

Management of palatally impacted canines requires surgical and orthodontic interventions. In the conventional method for managing impacted maxillary canines, comprehensive orthodontic treatment with fixed appliances is carried out. First, teeth are aligned and then a relatively stiff rectangular arch wire is inserted to minimize undesirable reactive movements of anchor teeth.^5^ However, this method may cause more root resorption of adjacent teeth during alignment and consolidation of anchor teeth, and may also lead to anchorage loss.^7^

Skeletal anchorage is required if we were to move only impacted teeth before fixed-appliance orthodontic treatment onset. TADs have become popular because of their ease of placement and removal, minimal need for patient compliance and relatively low cost.^9^ The advantages of TADs are that they remain relatively stationary in the bone, they are able to increase anchorage capacity and have no adverse effects or complications that could hinder health or treatment outcomes.^10^ Also, they facilitate difficult orthodontic tooth movements.^11^ ^,^ ^12^ Koscis and Seres^13^ suggested that miniscrew anchorage should be taken into consideration when extrusion of an impacted canine is planned.

The aim of this study was to use TADs to palatally move impacted canines away from the root of neighboring teeth before bracket placement, and to compare both the amount of root resorption and clinical success rate to the conventional procedure.

## MATERIAL AND METHODS

This was a non-randomized parallel-designed clinical controlled trial study. It was first performed as a pilot study on four patients and, based on the results, sample size was calculated (at least 11 patients for each group; α = 5% and power = 80%, effect size = 1.920). A total of 26 patients (15 in the experimental group and 11 in the control group) with 34 palatally impacted canines participated in this study. The experimental group consisted of 19 palatally impacted canines while the control group consisted of 15 palatally impacted canines. Patients were screened by panoramic radiograph, and impacted canines with axial inclinations < 45° were included in this study. Exclusion criteria were: history of orthodontic treatment, systemic disease, labially impacted canines and lack of proximity of canine and lateral incisor. All patients were females with a mean age of 15.6 ± 2.1 years old, and they all filled out and signed an informed consent form. The consent form was also signed by patients' parents. This study was approved by the Ethical Committee of Mashhad University of Medical Sciences (# 92/27712).

**Figure 1:**
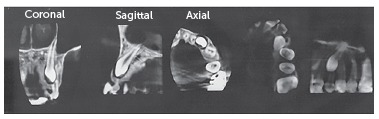
Pretreatment CBCT image.

**Figure 2:**
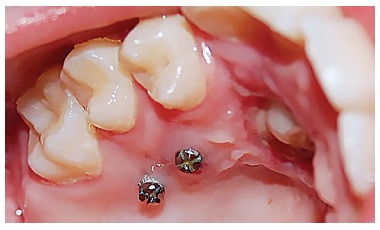
Miniscrews were inserted mesial and distal to the maxillary second premolar.

CBCT scans (Planmeca, Promax 3D Max, Helsinki, Finland) were taken from all patients (Fig 1). The scans were evaluated by an expert radiologist, and if the canine tooth was not in close approximation to lateral incisors root, the case was excluded from the study. After a thorough assessment of the experimental group, two miniscrews (Jeil, Seoul, South Korea) were inserted in the palatal region for each impacted tooth: one between the first and second premolar and another between the second premolar and first molar (Fig 2).

**Figure 3:**
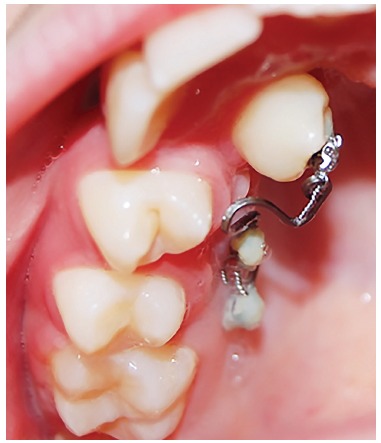
Four months after initiation of force application.

Miniscrews were of a bracket type 1.4 mm in diameter and 8 mm in length. The insertion site of the miniscrews was 5 mm from the embrasure; right angle to the palate. Two miniscrews were placed for anchorage reinforcement. Patients were then referred to a periodontist for surgical exposure of the impacted canine. After 10 days, periodontal dressing was removed and a bracket was bonded to the exposed surface of the canine. Subsequently, a 50-g force was applied to the bracket through a palatal cantilever spring made of 0.017 x 0.025-in TMA wire (Ormco, Glendora, California, USA). The cantilever spring was inserted into the slot of miniscrews 0.018 x 0.025-in and ligated with ligature wire. Miniscrews were covered by flowable composite resin. Every three weeks, force was adjusted until the canine erupted into the oral cavity (Fig 3). The miniscrews were then removed and comprehensive fixed orthodontic treatment began (Roth prescription 0.018-in slot Dentaurum, Pforzheim, Germany). After leveling and alignment were carried out with NiTi wires and sufficient space was gained, the erupted canine was guided to the line of occlusion using NiTi overlay. Patient's pain experience was measured by means of VAS (0 to 10) three weeks after initial loading and at the end of disimpaction treatment. 

At the end of treatment, another CBCT scan was taken to evaluate the resorption of canine and lateral incisors. Moreover, gingival index (GI) and bleeding on probing (BOP) were recorded for the erupted canine. Unerupted canines were reported as failure, and clinical success rate was calculated. The percentage of stable miniscrews was reported as survival rate of miniscrews. 

In the control group, comprehensive orthodontic treatment with fixed appliances was initially performed (Roth prescription 0.018-in slot, Dentauram, Germany). After initial leveling and alignment with NiTi wires, and after sufficient space was gained, a 0.016 x 0.022-in stainless steel arch wire was inserted and a transpalatal arch (TPA) was placed. Then the palatally impacted canine was erupted into the oral cavity by a cantilever spring made of 0.016 x 0.022-in stainless steel soldered to the palatal bar. This method was considered to be the safest method for disimpaction of palatally impacted canines.^14^ The erupted canine was guided to the arch with the aid of NiTi overlay. In both groups, the direction of force was initially away from the lateral incisors root. 

### Root resorption measurement

All CBCT scans were obtained by the same device at the following settings: exposures were made with 7 mA and 88 kV; and exposure time was of 12 seconds with a vowel size of 0.1 mm.

DICOM data sets of patients were imported into Amira software (Visage Imaging, Berlin, Germany). This software manually segments tissues according to Hounsfield units (HU). CBCT data were reconstructed with surface and volume rendering, and the volumetric image was manipulated to display the teeth from various orientations. Threshold values were set individually for each patient. The same HU were used for segmentation. On these 3D images, lateral incisor and canine were segmented. After segmentation, lateral incisor and canine were separated from other teeth, the volume of each tooth was measured and the two measurements (tooth volume loss and percentage of teeth volume loss) were calculated. Tooth volume loss was the difference between pretreatment (T_0_) and post-treatment (T_1_) tooth volumes. To calculate the method error, the volume of five canines and five lateral incisors was measured again by the same radiologist (r = 0.9, *p* = 0.001).

The radiologist who measured the volume of root resorption was blinded to the study. Data were analyzed by independent t-test, paired t-test and Mann-Whitney test (α = 0.05).

## RESULTS

After a three-week period, patients in the control group experienced higher pain levels than in the experimental group (*p* = 0.012); but, at the end of treatment, this difference was not statistically significant (*p* = 0.769). Moreover, in the experimental group, pain level was determined one day after placement of miniscrews, and mean value was 2.1 at this point in time. 

Descriptive statistics and comparison of tooth volumes between control and experimental groups are given in Table 1. For both canine and lateral incisors, the mean root volumes decreased from T_0_ to T_1_.

**Table 1:** Tooth volume (mm^3^) of canine and lateral incisor from T_0_ to T_1_.

Group	Tooth	Time	Mean	n	SD	T-test result
Test	Canine	T_0_	449.1958	19	20.49983	0.000
		T_1_	447.1368	19	21.20202	
	Lateral	T_0_	265.5984	19	21.43632	0.000
		T_1_	264.0774	19	21.32348	
Control	Canine	T_0_	456.1160	15	14.77856	0.000
		T_1_	454.3047	15	14.39192	
	Lateral	T_0_	265.2040	15	12.16210	0.000
		T_1_	259.2980	15	11.89779	

The volume of canine root resorption between control and experimental groups showed no statistically significant difference. However, the volume of lateral incisor root resorption in the control group was significantly greater than in the experimental group (nearly four-fold), as shown in Table 2. Gingival index of erupted canines did not show statistically significant difference between the two groups (*p* = 0.937). BOP test was also performed for erupted canines and it was not significant different between the two groups (Table 3).

**Table 2:** Comparison of the volume of root resorption in both groups.

Tooth	Group	n	Mean	SD	T-test result
Volume of canine root resorption (mm^3)^	Test	19	2.0589	1.34270	0.561
	Control	15	1.8113	1.04491	
Volume of lateral root resorption (mm^3)^	Test	19	1.5211	0.88833	0.000
	Control	15	5.9060	3.10025	
Percentage of lateral root resorption (%)	Test	19	0.0057	0.00343	0.000
	Control	15	0.0222	0.01160	
Percentage of canine root resorption (%)	Test	19	0.0047	0.00309	0.459
	Control	15	0.0039	0.00222	

**Table 3:** Bleeding on probing (BOP).

			BOP		Total
			Yes	No	
Group	Test	n	4	15	19
		%	21.1%	78.9%	100.0%
	Control	n	4	11	15
		%	26.7%	73.3%	100.0%
Total		n	8	26	34
		%	23.5%	76.5%	100.0%
Test result	*p* = 0.702		X^2^ = 0.147		

The mean duration of forced eruption was 5.2 months in the control group and 5.1 months in the experimental group, with no statistically significant difference between these two groups (*p* = 0.125).

In both groups, all impacted canines erupted into the oral cavity; therefore, clinical success rate was 100%. In the experimental group, two out of 38 miniscrews failed and were replaced. Therefore, survival rate was 94.7%.

## DISCUSSION

Palatally impacted canines are a clinical problem frequently encountered.^10^ Impacted canines have definite complications, such as root resorption on adjacent lateral incisors, and their disimpaction requires special techniques of which many have certain disadvantages. The traditional technique requires initial alignment and placement of heavy rectangular base arch wires to neutralize reaction forces.^5^ Although this approach is commonly used, it has several disadvantages. First, placement of brackets on the adjacent lateral incisor may lead its apex to be closer to the resorptive follicle of the impacted canine. Second, when rectangular wires are inserted, torque is expressed and it may cause further resorption of adjacent lateral incisors. Third, this process is time consuming, while the resorptive follicle of the impacted canine remains active.^7^ It seems logical in clinical practice to move impacted canines away from the roots of adjacent teeth before comprehensive arch orthodontic setup. Therefore, we decided to evaluate the effect of canine disimpaction before initiation of comprehensive orthodontic treatment on the root resorption of adjacent teeth in comparison to the aforementioned conventional technique. Disimpaction of canine without the aid of neighboring teeth require bone anchorage; therefore, we used two miniscrews to provide anchorage for canine eruption.^15^

In this study, GI, BOP, volumetric root resorption and success rate in the experimental group were compared to the traditional technique group in which, after anchorage preparation, we used a transpalatal arch to bring impacted teeth to the dental arch.

In both groups, patients' age (*p* = 0.625) and initial tooth volumes (canine *p* = 0.28; lateral incisor *p* = 0.947) were comparable, and the axial inclination of all canines was < 45°. Moreover, the results of the study showed no significant difference in mean duration of canine forced eruption between the two groups (*p* = 0.856).

Hu et al^16^ showed that TADs will not cause discomfort and pain during placement and treatment. This study confirms our findings. TADs mechanics is entirely based on their stability. Many studies have shown that the survival rate of TADs is greater than 80%.^17^ ^,^ ^18^ ^,^ ^19^ In our study, two of the 38 TADs failed (survival rate = 94.7%). All TADs in our study were placed in the palate, which may explain the higher survival rate.

Root resorption after orthodontic treatment has been evaluated by different devices, such as conventional radiograph and light microscopy or electron microscopy;^20,21^ although conventional 2D radiograph has many limitations for revealing root resorption.^22^ ^,^ ^23^ Chan and Darendeliler^24^ concluded that 2D radiographs are good diagnostic tools; however, quantitative evaluation should be avoided. An alternative to 2D radiograph is CBCT which is particularly useful in the evaluation of root resorption after orthodontic treatment; its nondistorted images allow thorough assessment of the root.^25^

CBCT gives us high-quality images with the same radiation dose of conventional radiograph. Only a few studies in the literature have measured tooth volume using CBCT. Wang et al^26^ compared the accuracy of CBCT for volumetric measurement of teeth by means of micro CT as the gold standard. They concluded that the accuracy of the CBCT method for volumetric measurement of teeth *in vivo* is comparable to the micro CT method *in vitro*. Therefore, the CBCT method has the potential to be applied in studies on root resorption associated with orthodontic treatment. Li et al^27^ showed volume measurement using CBCT which was able to evaluate root resorption caused by miniscrews intrusion.^27^ Walker et al^28^ showed that 3D techniques are more sensitive than 2D techniques.

In the current study, total tooth volumes were calculated based on CBCT scans. Tridimensional reconstruction of the tooth allowed us to study volume loss. Mean volumes of canine and lateral incisors were not significantly different at T_0_; thus, both groups were homogenous before treatment. After treatment, resorption of canines did not have statistically significant difference between groups. However, resorption of lateral teeth was almost four times greater in the control group than in the experimental group (*p* < 0.0001). In the control group, tooth alignments were performed first, which might have been responsible for further root resorption.

In this study, mean root volume was found to decrease in all examined teeth within groups from to T_0_ to T_1_. This fact may be due to the effect of orthodontic treatment on root resorption.

In the control group, tooth alignment was performed before canine forced eruption; however, in the experimental group, initially, canines moved away from the root of neighboring teeth due to our technique, which may explain significantly lower lateral root resorption. It seems that guiding palatally impacted canine away from the root of lateral incisors before bracket placement on other teeth is essential in impacted canine treatment.

Oberoie et al^29^ evaluated root resorption of the lateral incisor adjacent to impacted canines. Qualitatively, 40.4% had no root resorption, 35.7% showed slight root resorption, 14.2% showed moderate resorption and 4% showed severe root resorption of the adjacent lateral incisor.

Due to concerns about oral hygiene and gingivitis caused by the presence of miniscrews, GI and BOP of impacted canines were determined after forced eruption and compared between the two groups. Results showed no significant difference between the two groups, and this may indicate that the presence of miniscrews will not lead to gingivitis. All impacted canines erupted into the oral cavity; thus, clinical success rate was 100%.

As the root is a 3D structure, using tridimensional imaging modalities to evaluate orthodontic root resorption is very useful. Although micro CT is the best tool, it cannot be used in clinical studies. We recommend using CBCT instead of 2D imaging techniques for evaluating orthodontic root resorption in clinical situations.

## CONCLUSION

Disimpaction of palatally impacted canines before alignment of teeth may decrease root resorption. This illustrates that the use of TADs allows a more controlled movement of the impacted tooth. Another advantage of this method is that the maxillary arch may not be bracketed until canine disimpaction, and ankylosis can be ruled out. Patients' pain experience measured by VAS score was not different between the two groups.
